# Characterization of mesenchymal stromal cell-mediated mitochondrial transfer to healthy and diseased intervertebral disc cells

**DOI:** 10.1371/journal.pone.0357296

**Published:** 2026-09-15

**Authors:** Ashley Cardenas, Noah Willett, Anthony Robayo, Jessica Berger, Blake Boadi, Chibuikem A. Ikwuegbuenyi, Ibrahim Hussain, Roger Härtl, Lawrence J. Bonassar

**Affiliations:** 1 Meinig School of Biomedical Engineering, Cornell University, Ithaca, New York, United States of America; 2 Department of Neurological Surgery, Och Spine at New York Presbyterian Hospital, Weill Cornell Medicine, New York, New York, United States of America; 3 Sibley School of Mechanical and Aerospace Engineering, Cornell University, Ithaca, New York, United States of America; Yarmouk University, JORDAN

## Abstract

Intradiscal injection of mesenchymal stromal cells has gained widespread interest for the treatment of intervertebral disc degeneration. Mesenchymal stromal cells have been shown to impart a therapeutic benefit on treated tissues through a variety of mechanisms including mitochondrial transfer. Mitochondrial transfer from mesenchymal stromal cells to diseased cells has been demonstrated, in numerous model systems, to increase the survival and function of recipient cells. To date there are few studies that have investigated mitochondrial transfer from mesenchymal stromal cells to intervertebral disc cells. The goal of this study is to characterize mitochondrial transfer from mesenchymal stromal cells to bovine and human intervertebral disc cells using confocal microscopy. Following a 24-hour coculture period, bovine mesenchymal stromal cells were shown to transfer mitochondria to bovine annulus fibrosus cells under both healthy and inflammatory conditions. In addition, human mesenchymal stromal cells were shown to transfer mitochondria to diseased, patient-derived intervertebral disc cells. Mitochondrial transfer quantity was found to increase under inflammatory stress conditions and with patient age, though functional consequences of this transfer remain to be determined. This study is the first to show mesenchymal stromal cells transfer mitochondria to bovine annulus fibrosus cells and patient derived intervertebral disc cells. We provide evidence that mitochondrial transfer is a relevant mechanism by which mesenchymal stromal cells communicate with intervertebral disc cells.

## Introduction

Globally, over 5% of individuals suffer from symptomatic intervertebral disc (IVD) degeneration [1]. Clinical manifestations of degenerative disc disease include axial and radicular pain with radiographic observations such as a hypointense disc, herniation, and/or instability [2,3]. The gold standard treatments for early-stage degeneration include pain management and surgical intervention. However, such interventions emphasize immediate pain relief and fail to restore the IVD to its native state. As a result, many individuals, up to 46% for cases of lumbar herniations, will continue to experience postoperative pain as the disease worsens [4].

Currently, much of the investigational work in spine therapeutics focuses on revitalizing diseased cells and reducing the levels of inflammation to promote endogenous repair of the disc. Mesenchymal stromal cells (MSCs) are of growing interest as they can be isolated from numerous sources (such as muscle, adipose tissue, and bone marrow) and various studies have demonstrated their analgesic ability. In the U.S., bone marrow derived MSCs are the most widely used cell population for clinical trials investigating intradiscal injections [5–7]. In one study, injection of autologous, bone marrow derived MSCs into the disc of 5 individuals exhibiting degenerative disc disease improved patient quality of life up to 4–6 years post injection [7]. In a similar study investigating intradiscal injection of allogenic bone marrow derived MSCs, treated patients experienced a reduction in reported pain and disability scores 3 years post-injection [5]. Although the former saw some reduction in disc protrusion size, neither study reported changes in disease score. While these studies are promising, the limited knowledge on MSC fate, function, and, consequently, effectiveness post-transplantation continues to hinder improvements and clinical translation.

Advances in preclinical studies have worked to better elucidate mechanisms by which MSCs promote pain relief and tissue repair. It is now well accepted that MSCs revitalize diseased cells and tissues primarily by releasing various growth factors, microRNAs, cytokines, and even whole organelles [8–10]. Among the most novel points of interest is mitochondrial transfer, in which MSCs donate their own mitochondria to diseased cells through cell fusion, gap junctions, extracellular vesicles (EVs), and filopodial extensions [11–13]. Mitochondrial transfer from MSCs has been reported in numerous model systems including mitochondrial encephalomyopathy, spinal cord injury, and heart failure. Evidence from these studies demonstrates that MSC-mitochondria provide a range of functional benefits including preventing and protecting recipient cells from oxidative stress, apoptosis, senescence, and mitochondrial membrane potential loss (ΔΨ_M_), which is necessary for adenosine triphosphate (ATP) production [13–15]. Although mitochondrial dysfunction is regarded as a critical driver in degenerative diseases, MSC-mediated mitochondrial transfer has not yet gained wide attention in IVD degeneration.

Previous work investigating mitochondria in diseased IVD cells has shown functional, morphological, and genetic alterations that point to dysfunction. In one study, mitochondrial mass of annulus fibrosus cells (AFCs) was demonstrated to decrease with IVD degeneration while oxygen consumption increased, likely indicating leakage of electrons within the electron transport chain (ETC) [16]. Additionally, it was found that diseased IVD cells exhibited abnormally shaped and darkened mitochondria with small, poorly defined cristae and osmiophilic inclusion bodies, signifying possible mitochondrial DNA mutations such as those present in mitochondrial-related diseases [16]. In another study, various genes were also shown to be differentially upregulated in degenerative AFCs such as solute carriers, dehydrogenases, and cytochromes, again indicating potentially leaky mitochondria [17]. Like AFCs, aged nucleus pulposus cells (NPCs) have also been observed to have a reduced respiratory capacity as well as altered mitochondrial morphology and number compared to healthy NPCs [18]. It is well known that abnormal reactive oxygen species production, senescence, and apoptosis occur during disc degeneration [19–22]. Unsurprisingly, such alterations in cell behavior have a direct association with mitochondrial dysfunction [19,23,24].

Despite mitochondria playing a critical role in disc degeneration, to date there are few studies that have explored MSC-mediated mitochondrial transfer in the context of the IVD [25]. Results show that MSC-mitochondria reduce reactive oxygen species (ROS) production, increase ΔΨ_M_, and ATP production, while preventing cell apoptosis in NPCs treated with the mitochondrial inhibitor, rotenone. Nevertheless, the relevance of MSC-mediated mitochondrial transfer in more physiologic models of disc degeneration remains to be determined. Elucidating the mechanism of mitochondrial transfer to IVD cells is critical to developing new methods of enhancing MSC-based IVD therapeutics. In fact, although much attention has been paid toward annular repair [26–29], the extent to which MSCs transfer mitochondria to AFCs and factors that regulate mitochondrial transfer to this population of IVD cells remains unknown. Interleukin-1β (IL-1β) is a pro-inflammatory cytokine that has been previously shown to be upregulated in degenerated IVDs [30]. Various studies have used IL-1β to model IVD degeneration in vitro and in situ [31–34]. Accordingly, the aims of this study were to 1) use confocal microscopy to characterize MSC-mediated mitochondrial transfer to primary bovine AFCs, 2) assess the effect of IL-1β stimulation on MSC-mediated mitochondrial transfer, and 3) assess mitochondrial transfer from MSCs to IVD cells isolated from diseased human tissue.

## Materials and methods

### Bovine cell isolation

All bovine samples were obtained from a local butcher post-mortem, thus no IACUC approval was required. As described previously [35,36], three to five IVDs were harvested from each of six neonatal bovine tails and the nucleus pulposus of each IVD was removed. The annulus fibrosus was then minced and digested in a Ham’s F-12 (ThermoFisher Scientific Ham’s F-12 (1x), Waltham, MA) media supplemented with antibiotic antimycotic solution (100 units/mL penicillin, 100 µg/mL streptomycin, and 0.25 µg/mL Gibco Amphotericin B) (1x AbAm, Corning, NY) and 0.3% wt./vol. collagenase type II (Worthington Biochemical Corporation, Lakewood, NJ) for 6 hours. AFCs were extracted from the digest solution using a 100 µm filter, seeded at 8,000 cells/cm^2^ in T150 flasks, and expanded at 37°C, 5% ${\text{CO}}_{\text{2}}$, and 20% ${\text{O}}_{\text{2}}$with IVD media consisting of Ham’s F-12 supplemented with 10% FBS (FBS, Gemeni Bio, West Sacramento, CA) and 1x AbAm. Passage 1 (P1) AFCs were then frozen at ~1 million cells/mL in Cryostor (CS10, BioLife Solutions, Bothell, WA) until further use.

Bovine bone marrow stromal cells (bMSCs) were harvested from the femoral trabecular bone of one neonatal bovid as previously described [37,38]. Briefly, trabecular bone explants were washed with heparin and 1x AbAm supplemented DMEM, after which the media was centrifuged at 1800 RPM. Following centrifugation, all pelleted cells were plated evenly onto three T150 flasks and expanded at 37°C, 5% ${\text{CO}}_{\text{2}}$, and 20% ${\text{O}}_{\text{2}}$with bMSC media consisting of phenol red-free DMEM supplemented with 1 g/L glucose, 2 ng/mL basic fibroblast growth factor (bFGF), 2 mM L-glutamine, 25 mM HEPES, 10% FBS, and 1x Abam. At P1, cells were transduced with the rLV.EF1.mCherry-Mito-9 (Clontech Takara Bio Europe, Saint-Germain-en-Laye, France) lentivirus with a multiplicity of infection (MOI) of 30 to visualize MSC mitochondria, following manufacturer recommendations [12,38]. mCherry bMSCs were seeded onto T150 flasks at 3,000 cells/cm^2^ and expanded to P5 in bMSC media. P5 bMSCs were then frozen at 500,000 cells/mL in CS10 until further use. Media for AFCs and bMSCs was changed every 2–3 days.

### Mitochondrial membrane potential assessment

P1 AFCs were thawed and replated onto culture flasks at a density of ~3,000 cells/cm^2^. At ~70–80% confluence P2 AFCs were lifted using 0.25% trypsin with 2.21 mM EDTA (Corning, NY) and replated onto glass bottom dishes (ibidi, Fitchburg, WI) at a cell density of 8,500 cells/cm^2^*.* After an overnight incubation (~14 hours), the media was exchanged as follows: control AFCs received a media change with IVD media and the treated AFCs received a media change with IVD media supplemented with 10 ng/mL of IL-1β (SinoBiological, Beijing, China) to model inflammation [31,39]. Cells were cultured for an additional 48 hours before being stained with 13 µM of the ΔΨ_M_ dependent dye, JC10 (AAT Bioquest, Pleasanton, CA), in Hams F-12 media supplemented with 20 mM HEPES and 0.02% F-127, following manufacturer recommendations. Cells were then imaged using a Zeiss LSM880 inverted confocal microscope (Ex/Em: 488/532 nm and Ex/Em: 561/606 nm). 10 random two-dimensional images were taken per well (n = 3 donors, k = 3 samples/donor). For quantification, the integrated density (ID, Area x Mean Grey Value) per cell of the red and green channels was determined using ImageJ. The ID ratio of red to green signal (IDR) was used for comparison.

### Bovine AFC-MSC cocultures

P1 AFCs were thawed and replated onto culture flasks at a density of 3,000 cells/cm^2^. At ~70−80% confluence P2 AFCs were lifted using 0.25% trypsin with 2.21 mM EDTA and replated onto chambered glass slides (ThermoFisher Scientific Nunc™ Lab-Tek™ II Chambered Coverglass, Waltham, MA) at a cell density of 17,000 cells/cm^2^*.* After an overnight incubation, the media was exchanged as follows: control AFCs received a media change with IVD media and the treated AFCs received a media change with IVD media supplemented with 10 ng/mL of IL-1β. To visualize AFC boundaries, after 24 hours, AFCs were rinsed twice with phosphate buffered saline supplemented with 1x AbAm (PBS), stained with 3 uM of Vibrant^TM^ CFDA SE (ThermoFisher Scientific, Waltham, MA) for 15 minutes in PBS, rinsed twice with PBS, and incubated at 37°C, 5% ${\text{CO}}_{\text{2}}$, and 20% ${\text{O}}_{\text{2}}$for a minimum of 30 minutes alone in media. P5 bMSCs were then thawed and added to the culture wells at 8,500 cells/cm^2^ in a 1:1 solution of AFC:bMSC media. 10ng/mL of IL-1β was added along with the MSCs for wells previously treated with IL-1β. Cells were co-cultured for 24 hours then imaged using Zeiss LSM880 inverted confocal microscope (n = 4 donors, k = 4−8 replicates/donor). In summary, AFCs were exposed to IL-1β for 24 hours prior to the start of coculture with the MSCs and for a further 24 hours during coculture, as IL-1β was supplemented during the MSC addition. For each chamber, 7−14 imaging fields (resolution:.21−.35 μm/pixel) containing at least one MSC were randomly chosen. All imaging fields contained 10−20 Z-stack slices. The same protocol was followed for pretreatment of MSCs (n = 2 donors, k = 2 replicates/donor) and AFCs (n = 2 donors, k = 2 replicates/donor). However, 10 ng/mL IL-1β was used to treat each cell type for 24 hours *prior* to and was not maintained during coculture. Each IL-1β treated cell type was cocultured with its nontreated counterpart. For MSC pretreatment, P5 MSC were plated at a cell density of 12,000 cells/cm^2^ and cultured for 24 hours before the addition of IL-1β. Subsequently, MSCs were lifted using 0.25% trypsin with 2.21 mM EDTA and added to non-IL-1β-treated AFC cultures. Figures demonstrate images of AFCs stained green (CFDA) and MSCs genetically expressing red mitochondria (rLV.EF1.mCherry-Mito-9). For ΔΨ_M_ measurement in the cocultures, cells were stained with 140 nM of MitoView 405 (MV405) in Hams F12 media supplemented with 20 mM HEPES for 30 minutes prior to imaging (Ex/Em: 405/444 nm).

### Human cell isolation

Degenerative human disc cells (hDCs) were obtained from patients undergoing transforaminal lumbar interbody fusion (TLIF) or cervical artificial disc replacement (ADR) for refractory clinical symptoms (see Table 1). Human disc collection was approved by the Weill Cornell Medicine Institutional Review Board (Protocol #: 19–04020103). All patient samples and data were collected with written consent in accordance with institutional review board approval. Participants were consented on paper or digitally with a REDCap eConsent. Minors were not consented. Recruitment began March 29, 2024, and ended on October 29, 2024. Following surgery, disc samples were placed in cold PBS supplemented with 2x AbAm (200 units/mL penicillin, 200 µg/mL streptomycin, and 0.5 µg/mL Gibco Amphotericin B) for transportation. Samples were further rinsed with PBS supplemented with 2x AbAm before mincing and culture in IVD media. Following a 48–72-hour tissue culture period, samples were digested in Ham’s F-12 media supplemented with 1x AbAm and 0.3% wt./vol. collagenase type II for 3–4 hours, as described above. Isolated hDCs (containing both NPCs and AFCs that were not distinguished) were extracted from the digest solution using a 100 μm filter, seeded between 15,000–54,000 cells/cm^2^ in 6 well plates, and expanded in monolayer culture with IVD media at 37°C, 5% ${\text{CO}}_{\text{2}}$, and 20% ${\text{O}}_{\text{2}}$. Following P0 expansion, cells were lifted using 0.25% trypsin with 2.21 mM EDTA, replated at ~3,000–5,000 cells/cm^2^, expanded to P2, and frozen in 1 mL of CS10 until further use. Human mesenchymal stromal cells (hMSCs) were purchased from RoosterBio and transduced with rLV.EF1.mCherry-Mito-9 at an MOI of 8, following manufacturer recommendations. P5 mCherry hMSCs were frozen at ~200,000 cells/mL in CS10. Expansion and transduction media were purchased from RoosterBio. Accutase was used to lift hMSCs following manufacturer recommendations (Stem Cell Technologies, Vancouver, Canada).

**Table 1:** Patient information.

Age	Sex	BMI	Modified Pfirrmann Grade	Surgery Type	Level	Ratio of Cells with Colocalization
27	M	29.2	7	TLIF	L5/S1	0.80
64	M	30.4	5, 4	TLIF	L3/4, L4/L5	0.93
55	M	30.1	7	ADR	C5/6, C6/7	0.89
59	F	22.3	8	TLIF	L3/4, L4/5	0.93
46	F	31.7	5, 4	ADR	C5/6, C6/7	0.89
42	M	25.6	6	ADR	C3/4	0.91
46	F	24.6	6	TLIF	L4/5	0.87
79	M	24.5	7, 4	TLIF-L3/4 Microdiscectomy-L2/3	L3/4, L2/3	0.88

TLIF = Transforaminal lumbar interbody fusion; ADR = Artificial disc replacement; BMI = Body mass index

#### Human IVD-MSC cocultures.

P2 hDCs were thawed and seeded into chambered cover slides at a cell density of 17,000 cells/cm^2^. After an overnight incubation period, media was changed using IVD media. Following a 24-hour incubation, hDCs were stained with CFDA and hMSCs were thawed and added to the cultures following the same outline described for the bovine AFC-bMSC coculture. There was no addition of IL-1β at any time during the human cocultures. Cocultures were imaged using a Zeiss LSM880 inverted confocal microscope (n = 8 donors, k = 2–3 samples/donor). Figures demonstrate images of hDCs stained green (CFDA) and hMSCs genetically expressing red mitochondria (rLV.EF1.mCherry-Mito-9). For senescence β-galactosidase (β-gal) staining, cocultures were maintained for another 24 hours after imaging before fixation and staining (n = 2, k = 2).

#### Senescence β-galactosidase staining and quantification.

β-gal staining was conducted on hDC/MSC cocultures following manufacture recommendations (Cell Signaling Technology, Danvers, MA). Briefly, the provided fixative and staining solutions were diluted to 1x using UltraPure^TM^ Distilled Water (Invitrogen, Grand Island, NY). To create the β-gal staining solution, 80 µL of solution A, 80 µL of solution B, and 400 µL of 20 mg/mL X-gal (dissolved in dimethyl sulfoxide) was added to 7.440 mL of the 1x staining solution. The pH of the final β-gal staining solution was adjusted to 6 using 1M NaOH. Prior to staining, cells were rinsed twice with Hanks Balanced Salt Solution (Thermofisher Scientific 1x HBSS, Waltham, MA) and fixed for 15 minutes at room temperature using the 1x diluted fixative solution. Following fixation, cells were rinsed twice with PBS and the staining solution was added to each well. Plates were covered with parafilm and incubated at 37°C in a dry incubator for 12 hours. After the incubation period, staining was confirmed, and cells were rinsed three times with PBS. A Keyence BZ-X810 Microscope was used to acquire 5 random, two-dimensional images for each well. The fluorescent channels containing the mCherry and CFDA signal were used to identify hDCs. All green cells without extensive mCherry fluorescence were considered hDCs. The brightfield channel was used to identify which hDCs had blue, β-gal staining. Image analysis was conducted by a single unblinded investigator.

### Mitochondrial transfer quantification

**Fig 1:**
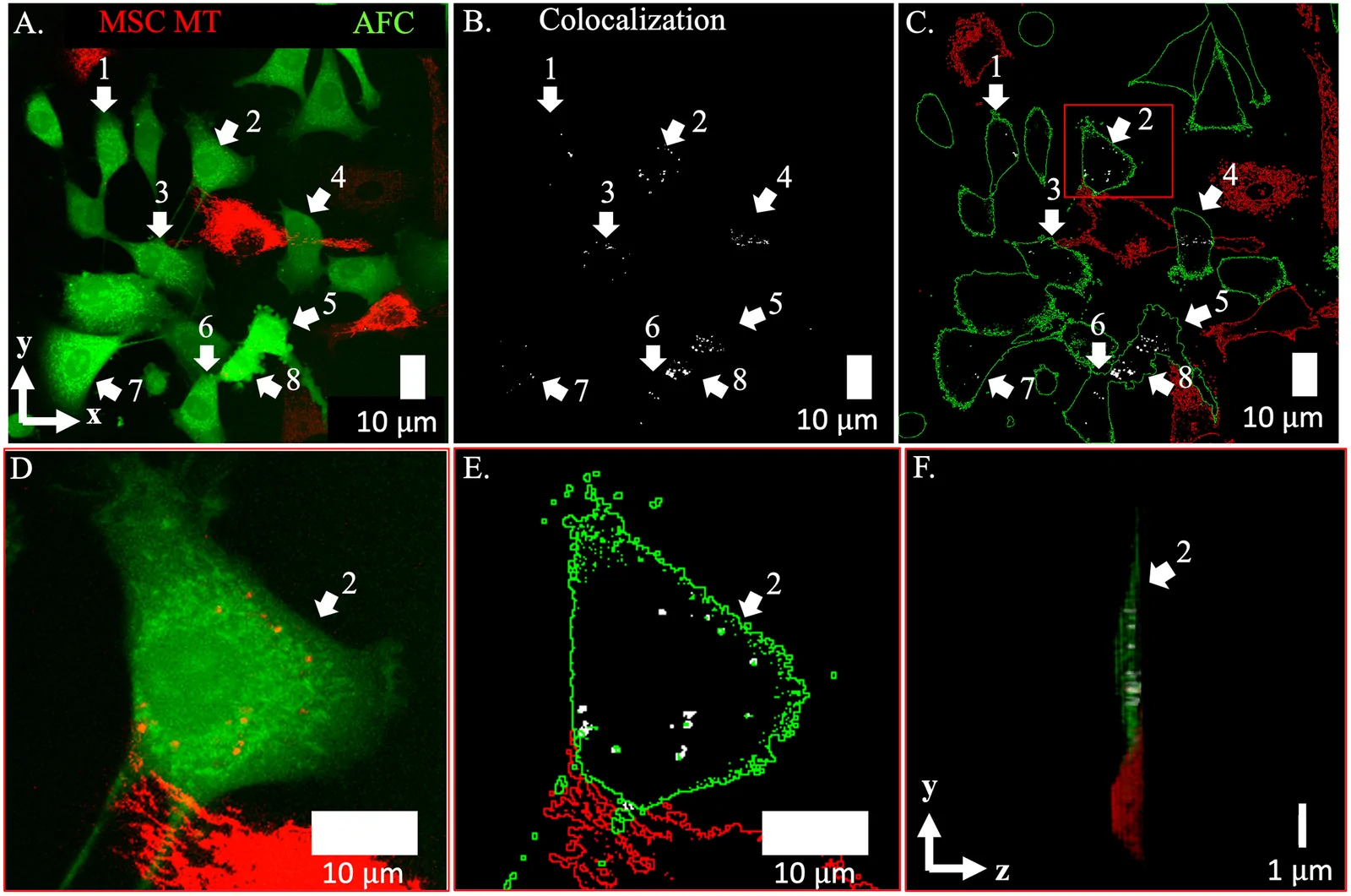
Quantification of mitochondrial transfer. Images A-C are collapsed Z-stacks with arrows pointing to signal colocalization. A.) Raw composite image. B.) Processed image containing all areas of signal colocalization. C.) Processed image containing outlined cells and signal colocalization. D.) Zoom-in of arrow 2 in raw, composite image. E.) Zoom-in of arrow 2 in processed image. F.) Orthogonal view of arrow 2 in processed image. Images A-E have 10 µm scale bars and F has a 1 µm scale bar.

Mitochondrial transfer events were identified by areas of colocalization between the CFDA (disc cells, ex/em: 488/527 nm) and mCherry (MSC mitochondria, ex/em: 561/616 nm) channels which were highlighted using an established ImageJ plugin (Fig 1) [40]. With the plugin, Isodata and Huang automatic thresholds were used to identify true signal from background in the CFDA and mCherry channels, respectively. Colocalization outputs were confirmed and counted manually using the raw images. All colocalized signals spanning more than one z-stack slice (.3 µm) and larger than one-pixel unit in area (.21 µm^2^) were considered a mitochondrial transfer event. Further, we note that it is possible for CFDA to leak out of AFCs and potentially be taken up by MSCs. Such cells would appear to have extensive mitochondrial networks and diffuse green signal. We observed very few such cells and excluded them from our mitochondrial transfer counts. The analysis of each colocalization signal on a per slice basis, in combination with our minimum size threshold, ensured that colocalization events were within the disc cell boundary and not a z-plane artifact in which mitochondria are above or below the cell (S2 Fig). Transfer events were only counted when the mCherry signal remained colocalized with CDFA positive AFCs across multiple consecutive z sections. We acknowledge that proximity artifacts cannot be completely excluded when using static confocal imaging approaches. However, this workflow was employed to reduce the possibility of mere signal overlap and to increase consistency in mitochondrial transfer quantification. A single cell with multiple colocalization signals was counted as one transfer event. All image analysis was conducted by the same unblinded investigator.

### Statistics

To determine differences in ΔΨ_M_, IDRs were log transformed, z-score normalized and a linear, nested mixed effects model was established in R-studio with random effects of donor, replicates within donor, and images within replicates. A Wald test was used for comparison between groups. Similarly, to determine the difference in mitochondrial transfer amount between the control and IL-1β AFC groups, a linear mixed effects model (LMM) was established in R-studio or Stata with donor as a random effect. In PRISM, an unpaired t-test was used to assess differences in the average mitochondrial transfer ratio between cells isolated from male and female patients as well as cervical and lumbar discs. With Stata, LMMs that assigned donor as a random effect were used to assess the relationship between patient age, Pfirrmann grade, and mitochondrial transfer ratio. Statistics were completed with the help of Cornell’s Statistical Consultation Unit. PRISM and Stata were used for graph creation. Bar graphs report mean ± standard deviation.

## Results

### MSCs transfer mitochondria to bovine AFCs

**Fig 2:**
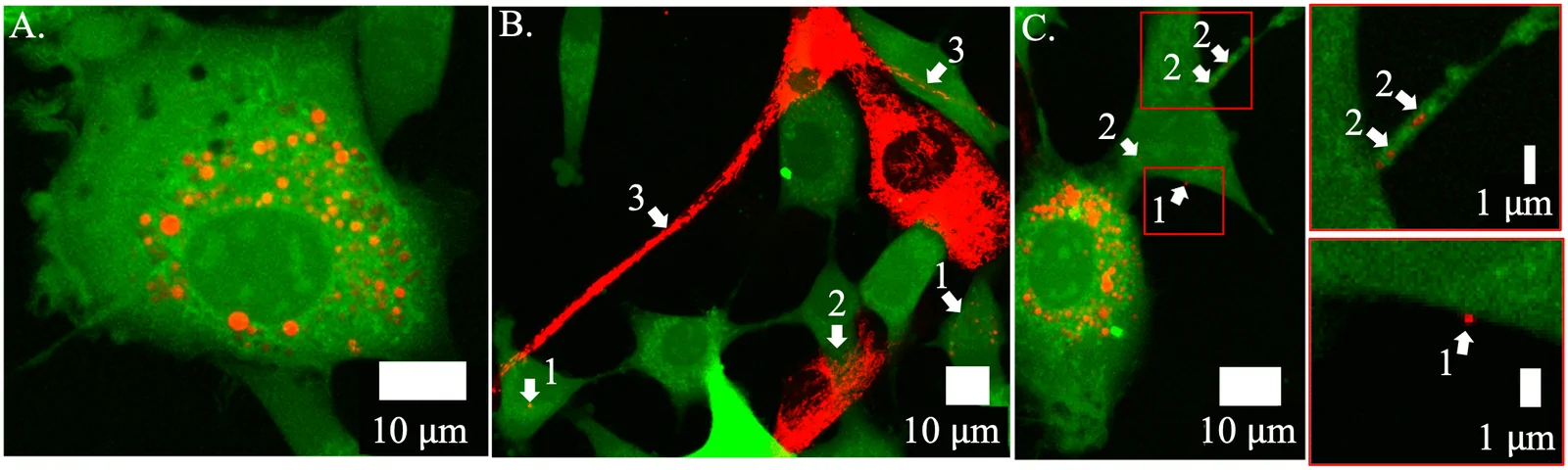
Mitochondrial transfer to Bovine AFCs. AFCs are green and MSC mitochondria are red. A.) AFC with multiple red mitochondria. B.) Evidence of internalized MSC mitochondria and active transfer. 1. AFC with few red mitochondria. 2. Fusion. 3. Filopodial extension. C.) 1. Red mitochondrion near an AFC (zoomed image is a single slice of z-stack). 2. Mitochondria within AFC extension. All images are collapsed z-stacks unless otherwise noted.

To determine whether bMSCs transfer mitochondria to healthy bovine AFCs, we conducted an AFC/MSC coculture in which AFCs were stained green and MSCs were genetically modified to express red mitochondria. Confocal images demonstrated AFCs with varying amounts of internalized red mitochondria (Fig 2A, B1). There was also evidence of mitochondrial transfer occurring through filopodial extensions (Fig. 2B3) and possible cell fusion (Fig. 2B2). Few cases were observed of red mitochondria near an AFC’s membrane (Fig. 2C1) and of mitochondria within AFC extensions (Fig. 2C2).

### MSCs transfer functional and non-functional mitochondria to bovine AFCs

**Fig 3:**
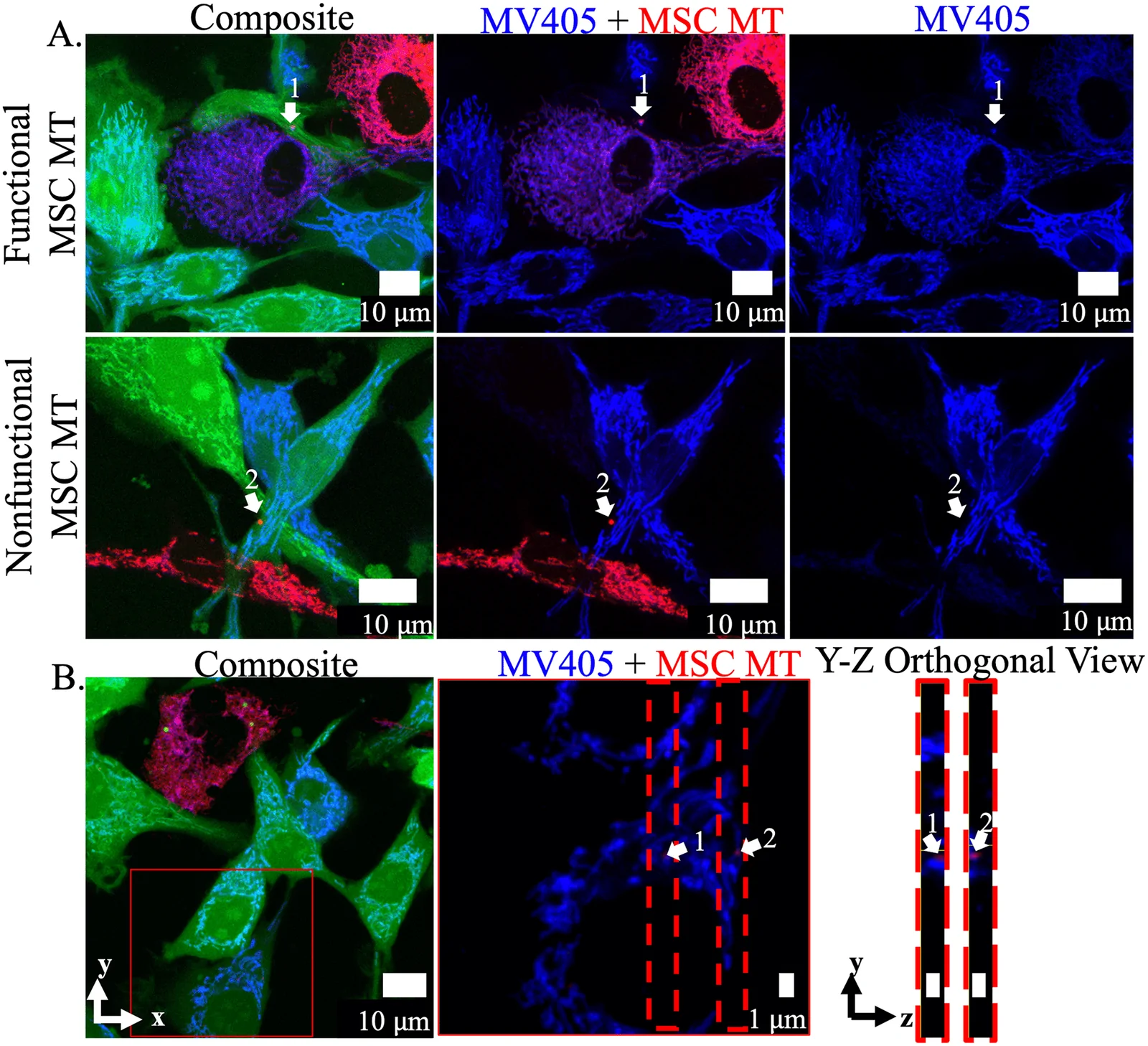
Functionality of transferred MSC mitochondria. Assessment of ΔΨ_M_ during AFC/MSC coculture. All images are collapsed z-stacks. AFCs are green and MSC mitochondria are red. All cells were stained with MV405. Bright blue fluorescence indicates polarized mitochondria. Arrows are pointing to transferred MSC mitochondria. A.) Top panel: AFC with a polarized MSC mitochondria. Bottom panel: AFC with a depolarized MSC mitochondria. B.) 1 and 2. Red mitochondria colocalizing with endogenous, blue AFC mitochondrial network.

Earlier findings indicate that MSC mitochondria can improve recipient cell respiration [12,25]. While it is believed that transferred mitochondria must incorporate into the recipient cell mitochondrial network to increase respiration, a recent study indicated that non-functional MSC mitochondria can also boost mitochondrial function [41,42]. Therefore, to investigate whether bMSCs were transferring functional or non-functional mitochondria, MV405 was added to MSC/AFC cocultures. MV405 is a membrane permeable fluorescent label that accumulates in polarized mitochondria producing brightly blue, fluorescent mitochondria. In mitochondria with disrupted membrane potential, MV405 diffuses out and into the cytoplasm. Confocal imaging demonstrated evidence of MSCs transferring polarized mitochondria (Fig 3A top panel) to an AFC with depolarized mitochondria and MSCs transferring depolarized mitochondria to AFCs with polarized mitochondria (Fig 3A bottom panel). Interestingly, there were also a few cases of MSC mitochondria colocalizing with the AFC mitochondrial network (Fig. 3B1, 2).

### IL-1β treatment triggers mitochondrial dysfunction in bovine AFCs

**Fig 4:**
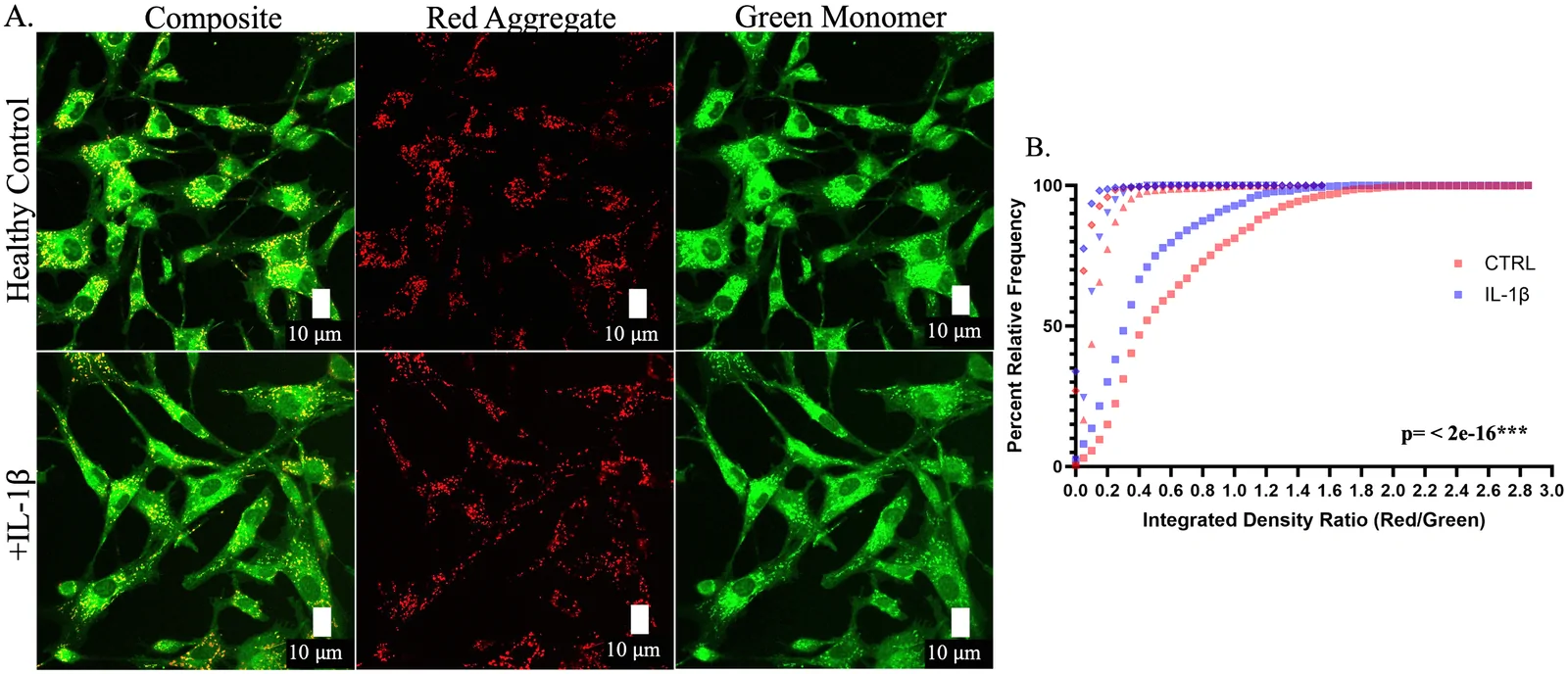
Effect of IL-1β treatment on AFC mitochondrial health. ΔΨ_M_ assessment in AFCs. Aggregate channel was changed to red for better visualization. All images were taken as singular optical slices. A.) Panels are showing red and green JC10 channels for AFCs after 48 hours of culture without IL-1β (healthy control, top panel) and + 10ng/mL of IL-1β (bottom panel). B.) Cumulative relative frequency of indicated IDRs (p=<2e-16). Higher IDRs are cells with more polarized mitochondria. Each symbol (i.e., square, triangle, diamond) represents a different donor animal.

Prior work demonstrated that direct inhibition of complex I in the mitochondrial ETC upregulates mitochondrial transfer from MSCs to dysfunctional cells [12,25]. It has been postulated that such increases in transfer are triggered by the release of mitochondrial components such as mitochondrial DNA and ROS from target dysfunctional cells, which act as environmental cues [8]. To determine whether physiologic mitochondrial stress could produce similar outcomes in AFCs, we used IL-1β, to promote an inflammatory phenotype [30]. To start, we wanted to determine to what extent IL-1β causes mitochondrial dysfunction in our model using JC10 to assess ΔΨ_M_. JC10 enters selectively into mitochondria and changes from green in its monomer form to orange in its aggregate form as ΔΨ_M_ increases, signifying healthy mitochondria. Visually, the amount of aggregate compared to monomer fluorescence was not substantially different between groups (Fig 4A). However, in the IDR relative frequency histograms of each donor animal (Fig 4B), there is a consistent leftward shift toward lower IDRs with IL-1β treatment, indicating that a higher percent of IL-1β treated cells had decreased ΔΨ_M_. Indeed, results showed that IL-1β treatment of AFCs caused an overall 28% decrease (95% CI: [25.1%, 31.3%]) in the IDR mean (0.09) compared to the healthy control (0.13) (Cohen’s d = −.40, p=<2e-16) (Fig 4, right). This data suggests that IL-1β treatment causes a consistent, small reduction in the number of polarized mitochondria per AFC, indicating a low level of mitochondrial dysfunction.

### IL-1β increases mitochondrial transfer to bovine AFCs

**Fig 5:**
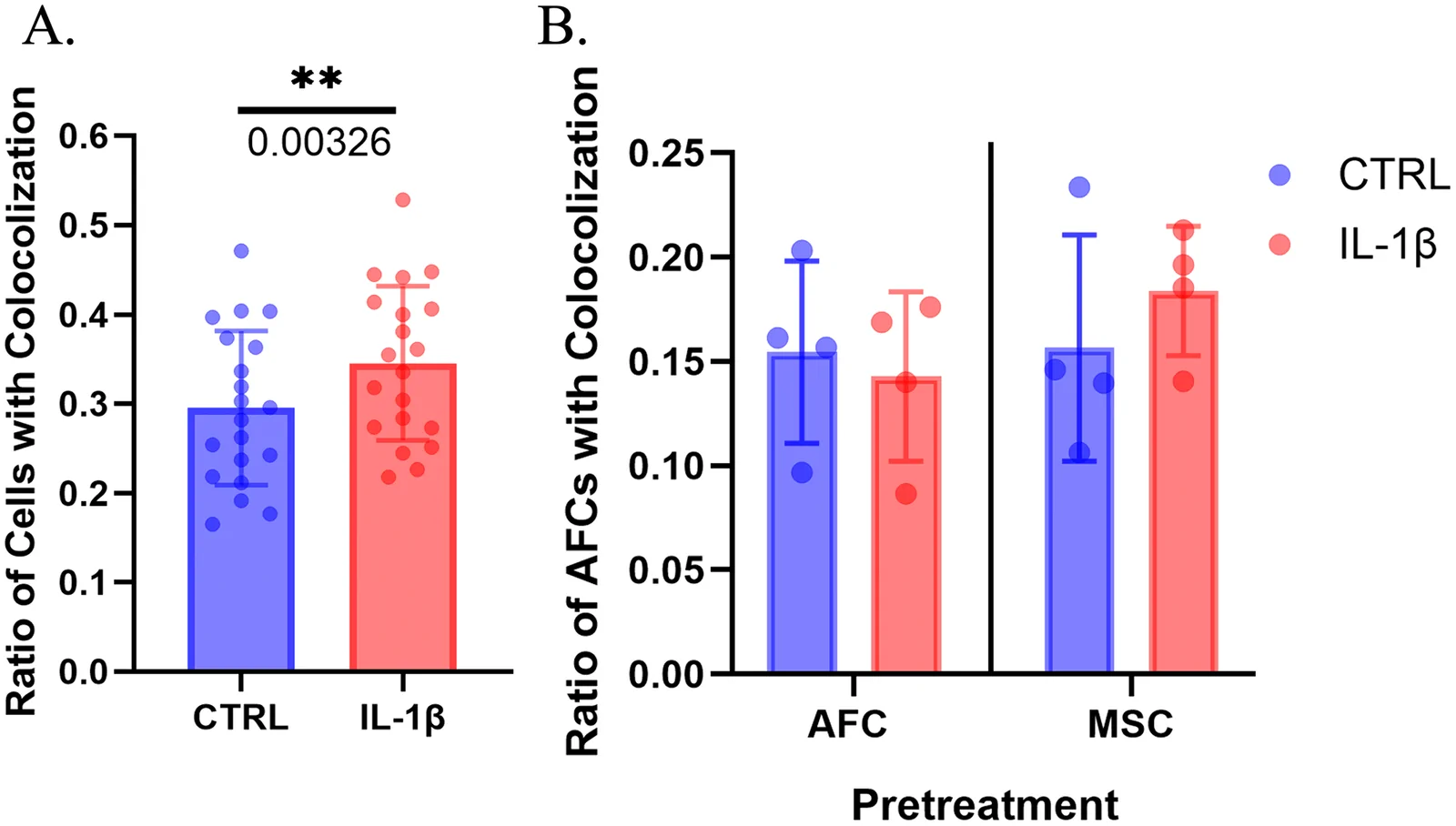
Number of AFCs with signal colocalization. A) IL-1β used to pretreat AFCs and maintained during AFC/MSC coculture (p = 0.00326). B) Left. Pretreatment of AFCs with IL-1β (p = .556). Right. Pretreatment of MSCs with IL-1β (p = .257). Graph reports mean ± standard deviation.

To determine whether IL-1β affects mitochondrial transfer, we conducted a bovine AFC/MSC coculture in the presence or absence of IL-1β. IL-1β increased mitochondrial transfer by 17% (Cohen’s d = 1.07, p = 0.00326), from a mean of 29% to 34% (representing an absolute increase of 5.01%, 95% CI: [1.79%, 8.23%]), in the overall number of cells with signal colocalization of the red MSC mitochondria and green AFC channels (Fig 5A). To determine whether IL-1β was driving transfer due to its effect on the AFCs or MSCs, each cell type was pretreated separately with IL-1β for 24 hours and cocultured with its untreated counterpart for an additional 24 hours. Pretreatment of AFCs decreased mitochondrial transfer from 15% in the nontreated, control AFCs to 14% in the IL-1β treated AFCs (p = .556) (Fig 5B left). Interestingly, pretreatment of MSCs increased mitochondrial transfer by 17%, from a mean of 16% in the control MSCs to 18% in the IL-1β treated MSCs (p = .257) (Fig 5B right).

### MSCs transfer mitochondria to human degenerative disc cells

**Fig 6:**
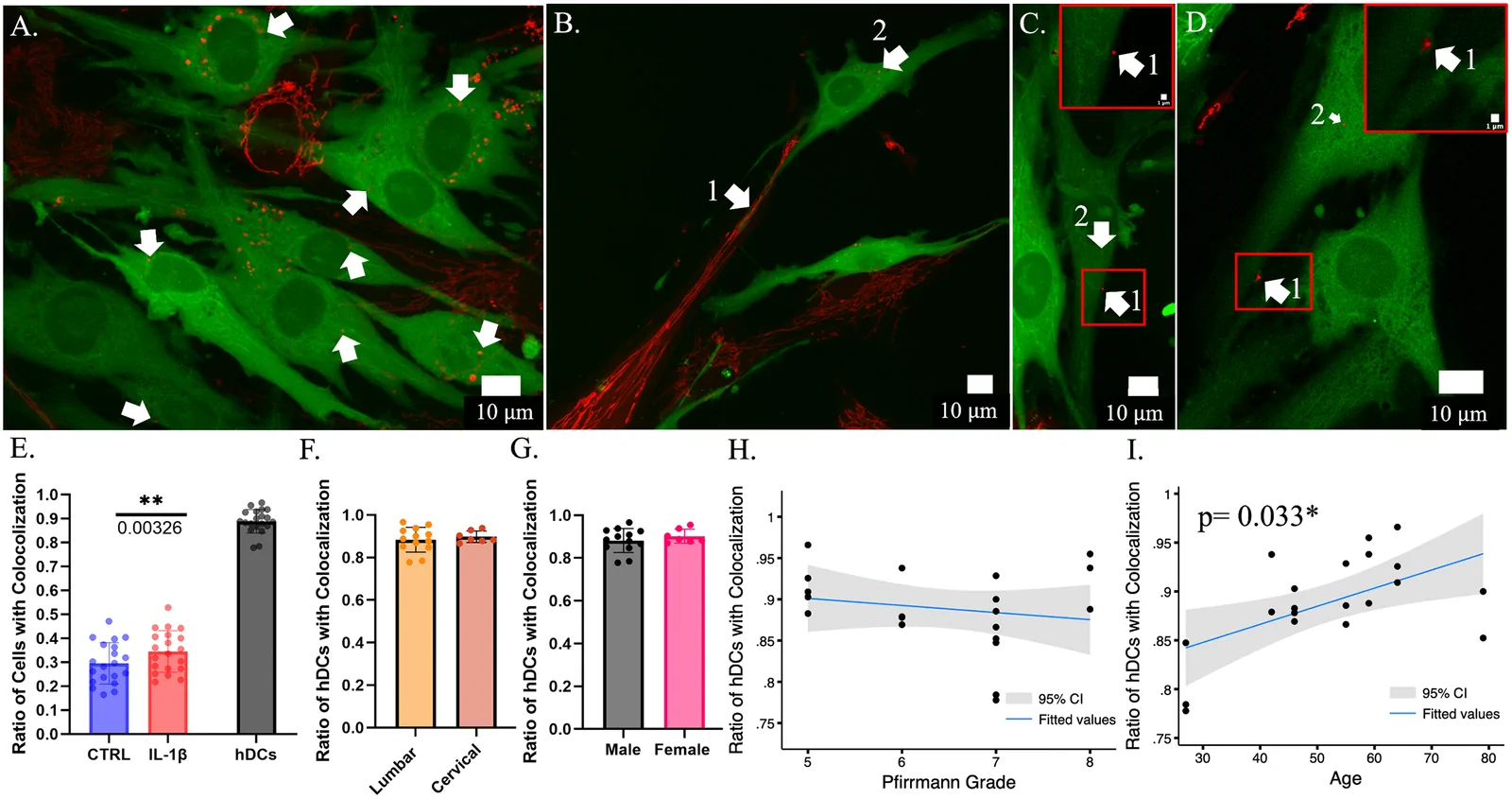
Mitochondrial transfer to hDCs. hDCs are green and hMSC mitochondria are red. All images are collapsed z-stacks. A) Evidence of hDCs with internalized MSC mitochondria. B) 1. Filopodial extension-mediated mitochondrial transfer. 2. Internalized red mitochondria. C) 1. Red mitochondria nearby an AFC membrane. 2. Internalized red mitochondria. D) 1. Live uptake of red mitochondria. 2. Internalized red mitochondria. E) Ratio of bovine AFCs and hDCs with signal colocalization. F) Ratio of hDCs with signal colocalization stratified by disc level (p = .550) G) Ratio of hDCs with signal colocalization stratified by sex (p = .654). Graph reports mean ± standard deviation. H) Patient Pfirrmann grade vs ratio of hDCs with signal colocalization (p = .0586). I) Patient age vs ratio of hDCs with signal colocalization (p = .033 by linear mixed-effects model).

To evaluate the importance of mitochondrial transfer in a physiologic disease state, human degenerative disc cells (hDCs) were isolated from patients undergoing surgery for the treatment of severe disc-degeneration. hMSC/hDC coculture (where hDCs were stained green and hMSCs were genetically modified to express red mitochondria) provided evidence of hDCs with internalized red mitochondria (Fig 6A, B2, C2, D2). Like the bovine cocultures, there was evidence of filopodial extension-mediated transfer (Fig. 6B1) and of red mitochondria near the membrane of some hDCs (Fig. 6C1). Confocal images also revealed apparent active uptake of red, extracellular mitochondria (Fig. 6D1). The amount of hDCs with red mitochondria was substantially higher than our observations with the bovine cocultures, with 89% of hDCs exhibiting at least one transferred mitochondrion (Fig 6E). No differences in mitochondrial transfer were found when patients were stratified by disc level (p = .550), sex (p = .654), or Pfirrmann grade (p = .0586) (Fig 6F-6H). However, the ratio of hDCs with transferred mitochondria was found to have a positive, linear relationship to patient age (p = .033). (Fig 6I).

### Effect of MSC treatment on senescence β-galactosidase staining in hDCs

**Fig 7:**
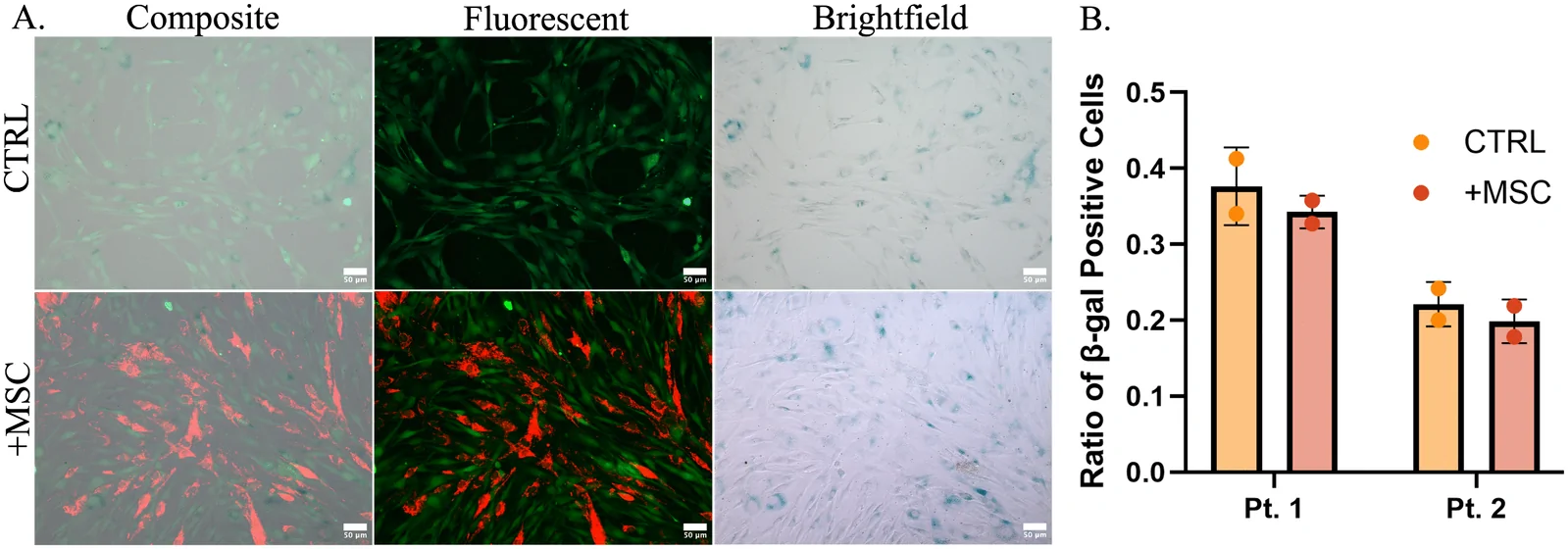
Quantification of β-gal in hDCs cultured alone vs with MSCs. A) hDCs are green and hMSC mitochondria are red. Panels show brightfield and fluorescent images of hDCs cultured alone (top) and with MSCs (bottom). B) Quantification of green hDCs with blue, β-gal staining for cells isolated from two patients (Pt 1. 46, F. Pt 2. 79, M).

To determine whether MSC coculture was having a positive effect on the recipient hDCs, senescence β-galactosidase staining was performed on the cocultures following 48 hours of incubation. Patient 1 demonstrated a baseline β gal positivity of 38% when cultured alone and 34% when cultured with MSCs (Fig. 7B). Similarly, Patient 2 demonstrated a baseline β gal positivity of 22% when cultured alone and 20% when cultured with MSCs (Fig. 7B). Across both patients we observe a nominal, 10% reduction in β gal-stained cells (Fig. 7).

## Discussion

The present study aimed to characterize MSC-mediated mitochondrial transfer to healthy, IL-1β-stressed, and diseased human IVD cells. The results from this study are the first to show that bovine MSCs transfer mitochondria to bovine AFCs under both healthy and inflammatory conditions, and that human MSCs transfer mitochondria to degenerative hDCs. Overall, the percentage of healthy bovine AFCs exhibiting a mitochondrial transfer event is similar to previous work showing that between ~15–30% of healthy cells will receive mitochondria from an MSC [41,43]. Interestingly, quantification of red and green colocalized signal suggests that mitochondrial transfer occurs to a greater extent under inflammatory conditions. Although this was statistically significant, it should be noted that the percent increase was modest (only 17%).

The resultant 17% increase in mitochondrial transfer following IL-1β treatment is much lower than what is reported in literature for cells treated with specific mitochondrial inhibitors [25,42,43]. Our mitochondrial transfer quantification method using confocal imaging and a fluorescent mitochondrial genetic tag is also unlike studies that use flow cytometry and/or mitochondrial dyes, which could contribute to the observed difference between our study and others. Nevertheless, this increase in mitochondrial transfer with the addition of IL-1β may indicate that inflammatory stimulation promotes mitochondrial transfer from MSCs or mitochondrial uptake by AFCs. Furthermore, pretreatment experiments demonstrated nonsignificant differences in transfer amounts when MSCs (16 → 18%) vs AFCs (14% → 18%) were pretreated with IL-1β. Although this may suggest that IL-1β has a direct stimulatory role on the MSCs themselves, these findings should be considered preliminary and hypothesis-generating rather than definitive, given the small sample size and lack of statistical significance. Accordingly, results from these experiments need verification with a larger sample size. Such observations do align with our JC10 results that show a modest 28% reduction in ΔΨ_M_ of AFCs with IL-1β treatment. As such this model represents early-stage or mild mitochondrial dysfunction rather than the severe bioenergetic failure observed in advanced degeneration. Whether mitochondrial transfer increases further in more severely dysfunctional cells remains to be determined. Additionally, maintenance of cultures under hyperoxic conditions (20% ${\text{O}}_{\text{2}}$) in this study may have affected both mitochondrial health and overall transfer dynamics, as previous literature shows hypoxia treated MSCs have improved mitochondrial health and overall regenerative potential compared to MSCs cultured under normoxic conditions [44]. Variations in the average control mitochondrial transfer ratio may be due to the use of MSCs that were expanded at different times and as such may have differences in overall activity.

Similar to IL-1β treatment, degenerative hDCs had a markedly large number of cells with red mitochondria, substantially surpassing numbers seen in bovine cocultures. While the exact mechanism remains unclear, such results are consistent with dysfunction-induced upregulation previously reported [45]. Due to the heterogenous nature of the human sample cohort and pooling of the mitochondrial transfer data, further analysis was conducted to determine whether any patient characteristics contributed to the mitochondrial transfer count differences observed between donors. Although we did not identify a relationship between mitochondrial transfer and donor patient sex, disc level, or Modified Pfirrmann Grade, our results did show an increase in mitochondrial transfer with age. Various factors such as senescence, mitochondrial function, and inflammatory signaling are also reported to be age dependent [46]. However, we are yet to identify which of these factors contribute to the suggested age dependency of mitochondrial transfer specifically. Future research with a larger sample size should also assess whether mitochondrial transfer frequency is affected by a specific combination of the reported donor characteristics. Furthermore, it may be possible that the amount of transfer was influenced by the cell composition of each sample (i.e., the amount of NPCs vs AFCs), given previous findings showing that MSCs preferentially transfer mitochondria depending on the cell type [45]. Unfortunately, we were unable to determine whether this was the case with NPCs and AFCs in this study as cells were pooled from both disc regions for all human samples and no cell marker analysis was performed. In addition, although cell expansion was minimized as much as possible, both hDCs and MSC may behave differently at earlier passages.

Confocal imaging of bovine cocultures indicated that MSCs transfer mitochondria to AFCs through various mechanisms. Filopodial extension and cell fusion were frequently observed transfer mechanisms. However, it is possible that mitochondria were also transferred through other mechanism such as EVs or gap junctions, which we did not directly identify in this study [11,12]. It is also likely that some of the reported filopodial extensions were tunneling nanotubes, an actin rich filopodial extension subtype [11]. All three mechanisms of transfer have been previously reported in other cell types. However, the dominating mechanism of transfer has been shown to be context dependent [13,47]. Although our study suggests that filopodial extensions and cell fusion are common, additional research should be conducted to determine which transfer mechanism predominates with IVD cells. Interestingly, findings of mitochondria within AFC extensions may indicate further movement of mitochondria among AFCs following transfer from MSCs and/or uptake of free floating or EV-encapsulated mitochondria from the surrounding environment. Similar mechanisms of transfer observed in bovine AFCs were seen in degenerative hDCs.

Assessment of ΔΨ_M_ following AFC/MSC coculture revealed that transferred mitochondria were both functional and non-functional. Prior work demonstrates that MSC mitochondria can fuse to the recipient cell mitochondrial network, enhancing respiration [42]. Such findings indicate that MSCs are donating functional mitochondria to stressed cells. However, non-functional mitochondria can also be beneficial to recipient cells through the mitophagy and mitochondrial biogenesis pathway [41]. For example, a recent study showed that artificial transplantation of non-functional MSC mitochondria improved endothelial cell respiration and engraftment by upregulating mitophagy via the pink1-parkin pathway, due to the presence of pink1 on donor MSC mitochondria [41]. Increased mitophagy subsequently lead to enhanced mitochondrial biogenesis, thus stimulating overall mitochondrial function in recipient cells [41]. Notably, these effects occurred despite the recipient cells having a competent mitochondrial network at baseline [41]. Our findings of functional and non-functional mitochondrial transfer, in addition to our observation consistent with MSC mitochondrial fusion into recipient mitochondrial networks, suggest that both mechanisms of respiration enhancement may be present in mildly stressed bovine AFCs. Although it’s unclear why certain cells received functional vs non-functional mitochondria in this study, given previous findings, it may be possible that the state of the recipient cell (healthy vs stressed) influences the prevailing mitochondria transferred. Future studies should work to elucidate the regenerative efficacy of functional vs non-functional mitochondrial transfer. Additionally, use of lysosomal markers would provide further insights as to the percentage of mitochondria that become integrated into the recipient cell network.

Quantification of β-gal staining in two patients showed nominal reductions with MSC coculture (38% → 34% and 22% → 20%). However, this observation is limited in sample size and does not provide clear evidence of functional benefit. While previous studies reported decreased β-gal staining following treatment with MSC secretome [48,49], our 48-hour total coculture may not have sufficient effect size to reveal a difference at this sample size. Importantly, this preliminary finding does not demonstrate that mitochondrial transfer improves diseased IVD cell function, hence the actual regenerative benefits of transfer on disc cells requires further assessment. Future studies with larger cohorts and additional functional assays (ATP production, oxygen consumption, apoptosis markers) are essential to establish therapeutic efficacy and mechanism.

Although we confirmed mitochondrial transfer between MSCs and both healthy and stressed IVD cells, there are limitations to this study. First, image analysis was unblinded, introducing the potential for observer bias. To mitigate this risk, we employed a standardized workflow using an ImageJ plugin and pre-defined minimum size threshold, as described in the methods section, to ensure a systematic and reproducible approach to image quantification. Furthermore, as with most imaging-based approaches, detection of small objects such as mitochondria can be influenced by the size threshold applied during image analysis. To mitigate errors in transfer quantification, we set our minimum size inclusion criteria to .21µ${\text{m}}^{\text{2}}$, which closely matches the diameter of MSC mitochondria that has been reported in literature to be 469±53nm [14]. MT transfer counts were insensitive to minimum size thresholds over the range of .15 to .3µ${\text{m}}^{\text{2}}$ (S1 Table). As such, we expect a low error rate in mitochondrial transfer quantification due to size-based filtering. However, it’s important to note that although we used a commercial mCherry lentivirus for mitochondrial visualization, we did not use mitochondria-specific markers such as TOM20 for further verification of mCherry localization within mitochondria. Furthermore, membrane staining of recipient cells would have provided supplementary evidence of mitochondria being fully internalized by recipient cells. An additional imaging related limitation to this study is the potential of our aged hDCs accumulating lipofuscin within their lysosomes. Lipofuscin is a fluorescent byproduct of degradation that exhibits a broad emission spectrum that overlaps that of mCherry [50,51]. To address this concern, we specifically imaged isolated hDCs and confirmed that there was no substantial background mCherry fluorescence (S1 Fig). Although with the aforementioned limitations, this method of quantification, using confocal microscopy, was chosen instead of flow cytometry to visualize and better characterize transfer events as well as prevent undercounting, since flow can be less sensitive to singular transfer events [52,53]. A remaining limitation to this study is that we did not provide any functional outcome measure to assess the specific effects of mitochondrial transfer on IVD cells. However, various other studies have shown the benefit MSC-mediated mitochondrial transfer has on the respiratory capacity, redox maintenance, and overall survival of target cells [25,41,54]. We also note that the samples size for our human cell cocultures is small (n = 8 donors, k = 2–3 replicates/donor). Although the observed trends reached statistical significance, further investigation with a larger cohort of samples is needed to confirm that these statistical trends hold for a wider population. Such studies have the potential of demonstrating that the relationship between mitochondrial transfer and patient age is non-linear. Similarly, all patient samples had relatively high Pfirrmann grades and as such widening the patient cohort may reveal a relationship between mitochondrial transfer and Pfirrmann grade that was not seen in this study.

## Conclusion

Our study shows that MSCs transfer both functional and non-functional mitochondria to healthy and stressed IVD cells. Furthermore, the amount of transfer was shown to be affected by both cell stress and aging. As such, our findings indicate that MSC-mediated mitochondrial transfer is a relevant mechanism in IVD applications. Further research needs to be conducted to determine 1) the functional impact of mitochondrial transfer on the health and survival of diseased IVD cells, 2) if mitochondrial transfer can result in tissue scale IVD regeneration, and 3) if enhancing transfer can increase the regenerative impact MSCs have on the IVD.

## Supporting information

S1 Fig Background fluorescence of hDCs. Images demonstrate collapsed z-stacks of hDCs cultured alone for 48 hours. Left. Composite image with both the CFDA and mCherry channels. Right. The mCherry channel alone. Top panel: 79-year-old. Bottom panel: 42-year-old.
(PNG)

S2 Fig Example MT transfer quantification. Images A-C are collapsed z-stacks of hDC/MSC cocultures. A.) Plugin output with ROIs > .21 highlighted in yellow. B.) >.21 ROIs overlayed onto raw image. C.) mCherry, MT channel for ROI referencing. D.) Top: Singular slice of z-stack. Yellow lines demonstrate specific y location on image. Bottom: x-z planes at the specified y locations.
(PNG)

S1 TableMitochondria minimum size threshold sensitivity analysis.Columns report the number of transfer events when the threshold is set to the listed sizes on ImageJ. .21μm is the baseline count as documented in the manuscript. Columns demonstrate little to no change in the transfer count when the size threshold is slightly decreased or increased to be within the documented size of a mitochondria.(PNG)
